# Theoretical study of the impact of adaptation on cell-fate heterogeneity and fractional killing

**DOI:** 10.1038/s41598-020-74238-y

**Published:** 2020-10-15

**Authors:** Julien Hurbain, Darka Labavić, Quentin Thommen, Benjamin Pfeuty

**Affiliations:** Univ. Lille, CNRS, UMR 8523 – PhLAM – Physique des Lasers Atomes et Molécules, 59000 Lille, France

**Keywords:** Cell death, Systems biology, Cellular noise, Differential equations, Multistability, Nonlinear dynamics, Regulatory networks, Stochastic modelling

## Abstract

Fractional killing illustrates the cell propensity to display a heterogeneous fate response over a wide range of stimuli. The interplay between the nonlinear and stochastic dynamics of biochemical networks plays a fundamental role in shaping this probabilistic response and in reconciling requirements for heterogeneity and controllability of cell-fate decisions. The stress-induced fate choice between life and death depends on an early adaptation response which may contribute to fractional killing by amplifying small differences between cells. To test this hypothesis, we consider a stochastic modeling framework suited for comprehensive sensitivity analysis of dose response curve through the computation of a fractionality index. Combining bifurcation analysis and Langevin simulation, we show that adaptation dynamics enhances noise-induced cell-fate heterogeneity by shifting from a saddle-node to a saddle-collision transition scenario. The generality of this result is further assessed by a computational analysis of a detailed regulatory network model of apoptosis initiation and by a theoretical analysis of stochastic bifurcation mechanisms. Overall, the present study identifies a cooperative interplay between stochastic, adaptation and decision intracellular processes that could promote cell-fate heterogeneity in many contexts.

## Introduction

In many adaptation and developmental contexts, isogenic cells make stochastic fate decisions to generate diversified cell types and subpopulations^1^. Cell-fate heterogeneity is indeed a key feature of microbial adaptation to adverse environments^2^, of the development and homeostasis of tissues and organs^3^ or of tumor resistance to drug therapy^4^. The differential fate outcome of isogenic cells exposed to the same environmental stimuli involves the interplay of stochastic and deterministic mechanisms^5–7^, where regulatory mechanisms can determine both the statistics and dynamics of stochastic events and the effect of those stochastic events on molecular trajectories dictating cell fate choices. Several experimental studies have shown that cell-fate decisions are often preceded by a highly fluctuating intracellular dynamics. Pulsatile or oscillatory activities have been observed in signaling pathways operating during the stochastic choice of various differentiation or proliferation fates^8–13^. The profile characteristics of those dynamic signaling activities have been proposed to either direct decision outcomes or promote cell-fate heterogeneity^14,15^. Transient dynamics occurring at epigenetics, transcriptome-wide or multicellular levels^16–18^ have also been proposed to regulate cell-fate heterogeneity and plasticity. All these examples support a key role of transient dynamics in orchestrating fate decisions from diverse signaling and stochastic cues.

An attractive case study is the stochastic fate decision between life and death, commonly termed fractional killing, for which the systematic measure of probabilistic dose-response curves coupled with single-cell analysis of stochastic and dynamical signatures are possible^19^. On this issue, several modelling studies have been devoted to identify which sources of fluctuations and which parts of the apoptotic network could contribute the most to the variability of decision time and outcomes^20–23^, while the impact of the transient dynamics has been seldomly addressed^24^. Yet, singe-cell analysis of the temporal trajectories of caspase 8 activity in response to TRAIL has revealed a signature of adaptation dynamics whose transient kinetics determines whether a cell survives or dies^25^. Caspase 8 is likely to be part of negative feedback regulation involving for instance the formation of inactive heterodimers of procaspase-8^26^. The importance of transient dynamics of apoptotic inducers has been also emphasized in the case of cisplatin drug exposure^24,27^. The proposed mechanism involves a competition between positive and negative regulation of caspase 8-dependent apoptosis, thereby defining an incoherent feedforward loop. More generally, environmental stressors are prone to upregulate both pro-survival and pro-death pathways^28–31^ through negative feedback or incoherent feedforward loop motifs which ultimately lead to a dynamical adaptation response^32–34^. These stochastic and deterministic features associated with fractional killing raise the more general question of the role of adaptation dynamics in shaping the timing and probabilities of stochastic fate decisions.

Diverse modelling approaches have proved useful to study stochastic switching in regulatory networks, ranging from the discrete chemical master equations and stochastic simulation algorithms to the continuous Fokker-Planck and Langevin equations. Those diverse tools and their refinements have been broadly used to study the interplay between noise properties and network topologies in shaping the steady-state bimodal distribution and transition rates associated with two phenotypic states^35–39^. In the present study, we use the joint framework of chemical Langevin equations^40^ and bifurcation theory to address the interplay of stochasticity, transient adaptation and bistable switching. The deterministic and stochastic analysis of a simple model combining adaptation and bistability deciphers how the adaptation overshoot dynamics modulate, concomitantly, the nonlinear decision-making properties and the probabilistic fate-response properties. The biological relevance of this behavior is assessed by simulating a more detailed model of programmed death pathways. Finally, the generality of the proposed noise-amplification mechanism is addressed within the theoretical framework of stochastic nonlinear dynamics.

## Results

### Stochastic modeling framework for probabilistic fate decisions

Fractional killing can be defined as the population-level property by which isogenic cells exposed to increasing doses of death-inducing stimuli will tend to display a fraction of surviving cells and dying cells, although with increasing probability of death. This stochastic decision process can be studied in a general theoretical framework that applies to cases of fate decisions other than survival and death. Probabilistic cell-fate response commonly involves the interplay between intracellular mechanisms of fate decision and intracellular sources of cell-to-cell variability. Without loss of generality, a possible framework to study such probabilistic fate response to a step stimulus consists in a Langevin differential equation description of the stochastic dynamics of a biochemical reaction network (see Table 1 for notations):

1a $$\begin{aligned} \frac{dx_i^l}{dt}= \sum _j \nu _{ji}\,a_{j}^l(t) + \sum _j \nu _{ji}\sqrt{a_{j}^l(t)}\,\xi _j^l(t) \end{aligned}$$

1b $$\begin{aligned} a_j^l(t)= a_{j}(\vec {x}^l,s^l(t),\vec {k}^l) \end{aligned}$$

1c $$\begin{aligned} s(t)= s\,H(t) \end{aligned}$$

1d $$\begin{aligned} \langle\xi _j^l(t)\,\xi _{j'}^{l'}(t')\rangle= \sigma ^2\,\delta (t-t')\delta _{j,j'}\delta _{l,l'} \,. \end{aligned}$$

where *H*(*t*) and $\delta (t)$ are respectively the Heaviside and the Dirac delta functions. In this model, the cell-to-cell variability of stimulus-induced response trajectories $\vec {x}^l$ can originate either from noisy biochemical reactions involving stochastic processes $\xi _j^l(t)$ or from heterogeneities in stimulus exposure/sensitivity $s^l$ or network parameters $\vec {k}^l$ from cell to cell. Although limited or inaccurate to describe some stochastic behaviors of biochemical networks^41^, chemical Langevin equation approach is nevertheless convenient to study asymptotic cases of small noise, large size or separated timescales and, thus, to relate with noise-free dynamical properties^42^.

**Table 1:** List of mathematical symbols and notations.

Symbol	Description	Equations/Figures
$x_i^l$, $\vec {x}^l$	Concentration of biochemical species *i* of cell *l*	Eq. (1)
$k_i^l$, $\vec {k}^l$	Biochemical network parameter *i* of cell *l*	Eq. (1)
$a_j$, $\nu _{ji}$	Rate and stoichiometries of the biochemical reaction *j*	Eq. (1)
$\xi _j^l$	Langevin noise associated to reaction *j* in cell *l*	Eq. (1)
$\sigma$	Standard deviation of random variable	Eq. (1)
$s^l$	Stimulus (e.g., stress) level of cell *l*	Eq. (1)
$s_{sn}$	Stimulus level associated with saddle-node bifurcation	Fig. 2
$P(\vec {x},t)$	Time-dependent probability distribution function in state space	Eq. (2)
$P_{D/Death}$	Decision (e.g., death) probability	Eq. (2)
$s_{50}$	Stimulus level inducing $50\%$ of fate probability	Eq. (3)
$t^*$	Measurement time for $P_D$	Eq. (2)
$\eta$	Fractionality index	Eq. (3)
$x_{1,2,3}$	Adaptive (e.g., damage/repair) and fate-decision (e.g., death) species	Eq. (4)
$\beta ,\tau$	Adaptation strength and timescale	Eq. (4)
$\vec {x}_{st1,2/sn/sad}$	Stable/saddle-node/saddle fixed point associated with bistability	Figs. 2, 3 and 4
$\mathcal{W}^{s/u}(\vec {x})$	Stable/unstable manifold of the fixed point $\vec {x}$	Eqs. (2) and (9)
$s_{c}$	Critical stimulus level without noise $s_c=s_{50}(\sigma =0)$	Fig. 2
$\vec {x}_c(t,s_c)$	Critical trajectory	Fig. 2 and Eqs. (7–8)
$\vec {y}(t)$, $y_N$	Small deviations of $\vec {x}(t)$ from $\vec {x}_c(t)$	Fig. 5 and Eqs. (7–9)
$\Pi (t,t')$	Principal fundamental matrix	Eq. (7)
$U(x),\Delta ,r_K$	Effective potential, barrier height and Kramers rate	Fig. 5 and Eq. (10)

**Figure 1:**
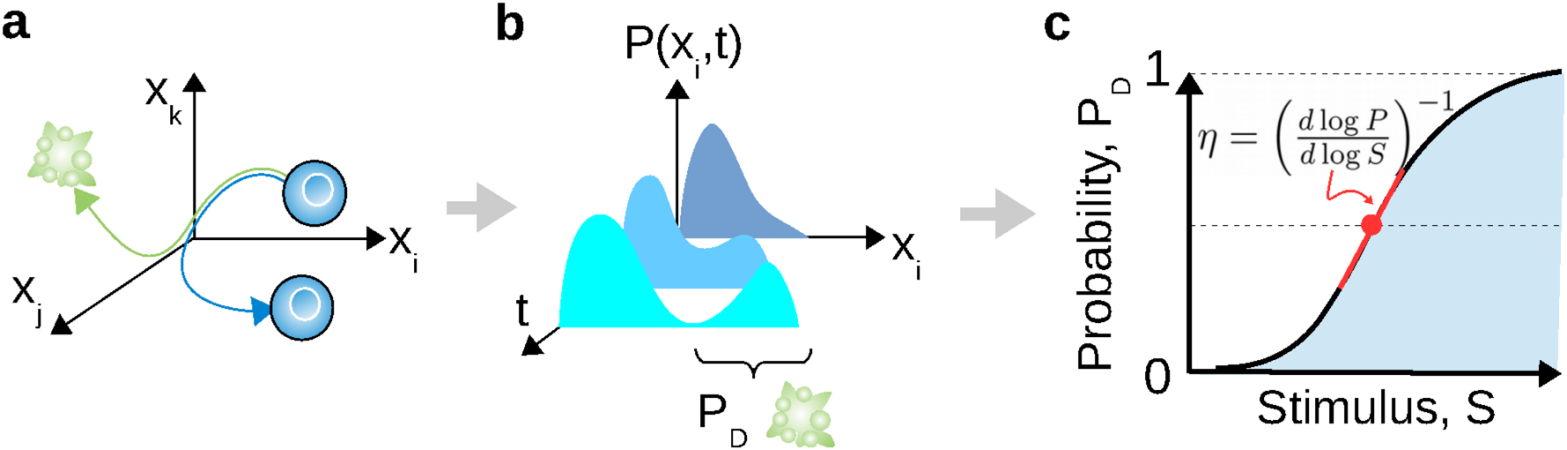
Dynamical and probabilistic schemes of cell-fate decisions. (**a**) State-space trajectories diverging toward distinct cellular phenotypic states. (**b**) Establishment of a bimodal probability density function. (**c**) Fate probability curves whose slope is quantified by a fractionality index ($\eta$).

For the deterministic part of the equation, the fate-decision behavior (e.g., death) can be minimally implemented in the nonlinear dynamics of the biochemical network by the presence of a saddle-node bifurcation mechanism at a critical stimulus level $s_{sn}$ through which the (survival) steady state $\vec {x}_{st1}$ is destabilized toward the other (death) steady state $\vec {x}_{st2}$, generally in an irreversible manner. Accordingly, near-identical cells exposed to the same stimulus may display divergent trajectories toward survival or death (Fig. 1a). The population dynamics of noisy or heterogeneous cells described by Eq. (1) can be statistically represented by a probability distribution $P(\vec {x},t)$, which typically follows a Fokker-Planck equation. Stimulus-induced fate decision relates with the establishment of a bimodal distribution such that one can define the decision probability $P_D$ (Fig. 1b):

2 $$\begin{aligned} P_{D}=\int _{\vec {x}\in \mathcal{W}^s(\vec {x}_{st2})}P(\vec {x},t^*)d\vec {x}\,. \end{aligned}$$

where $t^*$ is the typical measurement time and $\mathcal{W}^s(\vec {x}_{st})$ is the fate attractor basin. It is to emphasize that we consider the typical case of an irreversible fate decision associated with a low or null probability to revert from the state $\vec{x}_{st2}$ to $\vec{x}_{st1}$, which is typically the case for death, proliferation or terminal differentiation fate outcomes. From the dose-response curve $P_{D}(s)$, one can define a *fractionality index* (Fig. 1c) which quantifies the derivative of this curve around the $50\%$ fate probability ($P_{D}(s_{50})=0.5$):

3 $$\begin{aligned} \eta =\left( \frac{d\ln {P}}{d\ln {s}}(s_{50})\right) ^{-1} \end{aligned}$$

Based on this simple sensitivity measure of the stochastic fate response, systematic analysis of how $\eta$ varies with noise strength $\sigma$ and network parameters $\vec {k}$ should provide key insights into the interplay of stochastic and nonlinear properties of networks in shaping probabilistic features of fate response.

### Adaptation dynamics enforces a saddle-collision mechanism for decision making

**Figure 2:**
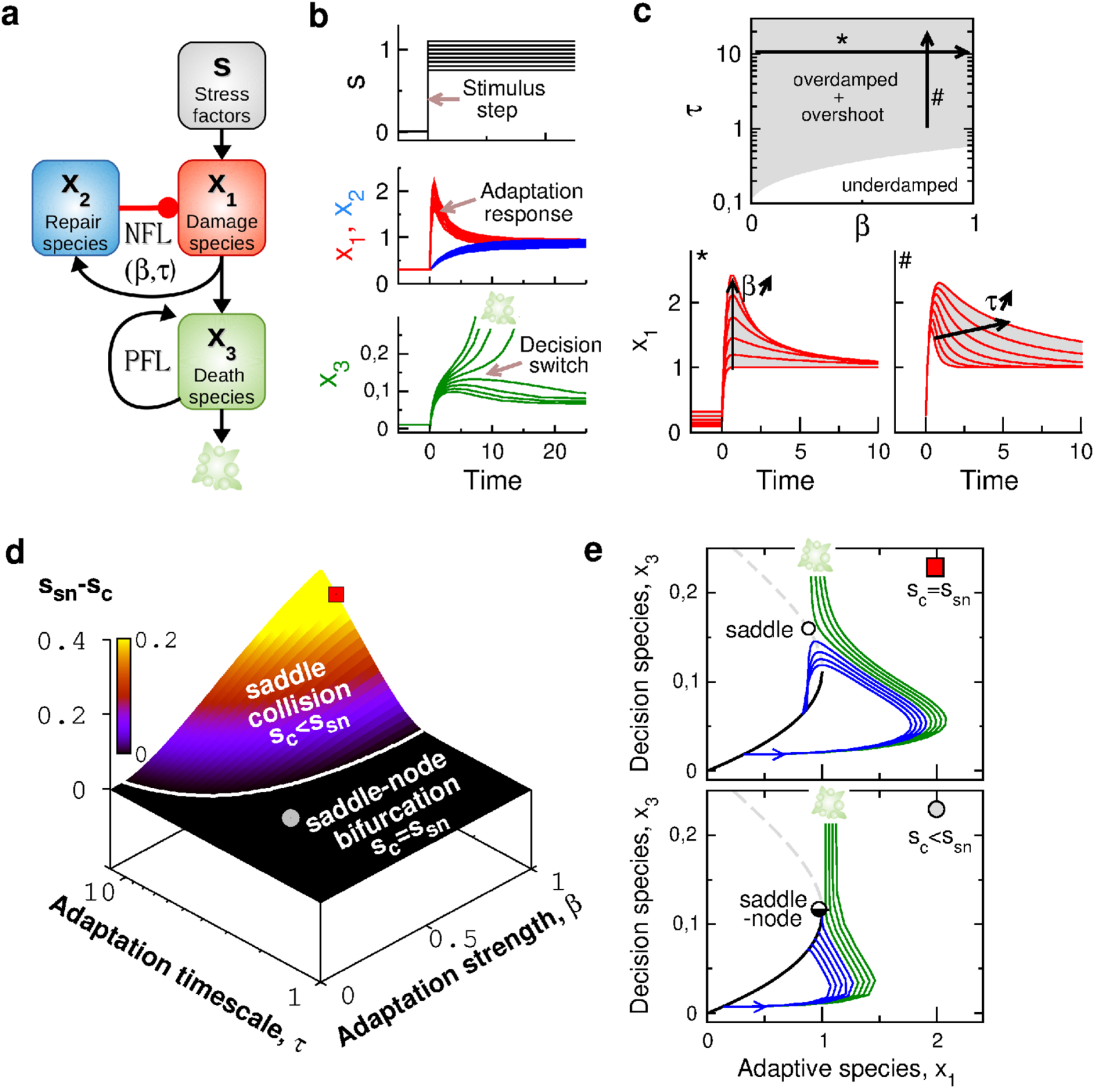
Adaptation alters the nonlinear mechanism of decision making. (**a**) Coarse grained model combining a negative feedback loop (NFL) between $x_1$ and $x_2$ species and self-activation positive feedback loop (PFL) of $x_3$ species. (**b**) Typical adaptation and switching dynamics in response to a stimulus step. Color code relates to that of panel (**a**) and model parameters are $\beta =1$ and $\tau =10$. (**c**) Effect of adaptation parameters $\beta$ and $\tau$ on the linear response regime (upper panel) and the overshoot profile of the adaptation response of $x_1(t)$ (left and right bottom panel). (**d**) Plot of $s_{sn}-s_{c}$ as a function of $\beta$ and $\tau$ where two distinct transition regimes ($s_c=s_{sn}$ and $s_c<s_{sn}$) are separated by the white boundary. (**e**) Single-cell trajectories plotted in the $\{x_1,x_3\}$ space for increasing level of stimulus *s* (blue for $s<s_c$ and green for $s>s_c$): Upper panel (red square: $\beta =1$ and $\tau =10$) shows a saddle collision for $s=s_c$ and bottom panel (grey circle: $\beta =0.3$; $\tau =3$) shows a saddle-node bifurcation. Black full and gray dashed lines represent the steady state branches $\vec {x}_{st1}(s)$ and $\vec {x}_{sad}(s)$.

To evaluate the impact of adaptation dynamics on the probabilistic properties of stochastic fate decisions, the biochemical reaction model used in Eq. (1) must implement adaptation and switching behaviors. For the ease of mathematical and graphical analysis, we consider a low-dimensional biochemical reaction network^43^, whose interactions between three coarse-grained variables implement a basic setting of a negative feedback-driven adaptation and a positive feedback-driven decision switch (Fig. 2a,b). Starting from a set of biochemical reactions associated with this architecture, a suitable factorization and normalization procedure (see "Methods" section) allows one to derive the following set of differential equation,

4a $$\begin{aligned} \tau _1 \frac{dx_1}{dt}&= 1- k_1 + k_1 \,s(t) - x_2\,x_1\,, \end{aligned}$$

4b $$\begin{aligned} \tau \frac{dx_2}{dt}&= 1-\beta + \beta x_1 - x_2 \,, \end{aligned}$$

4c $$\begin{aligned} \frac{dx_3}{dt}&= k_2 \,x_1 + k_3\,\frac{x_3^2}{ k_4 + x_3^2} - x_3 \,. \end{aligned}$$

The reaction rates $a_i$ of the corresponding Langevin equations are detailed in "Methods" section. Most parameter values are fixed within a range consistent with some biological assumptions. First, self-activation parameters $k_3=1$ and $k_4=0.2$ implement a positive feedback that is strong enough to produce an irreversible transition to death fate. Second, the parameters $k_1=0.9$ and $\tau _1=0.1<1<\tau$ implement a significant and fast stimulus-induced response of $x_1$, which gives rise to a marked overshoot dynamics through negative feedback with $x_2$. Third, the synthesis rate parameters $1-\beta$, $1-k_1$ and $k_2=0.056$ satisfy the normalization condition that saddle-node bifurcation occurs for $x_1=x_2=s=1$ whatever the values of the other model parameters ($k_i$, $\beta$, $\tau$).

In this way, the parameters $\beta$ ($\in [0:1]$) and $\tau$ can be systematically varied to modulate the overshoot characteristics of adaptation with limited impact on steady-state properties. Indeed, the steady state of $x_1$ depends on the stimulus according to:

5 $$\begin{aligned} x_1(s)=\frac{\beta -1 + \sqrt{(1-\beta )^2 + 4 (1-k_1+k_1 s)\,\beta }}{2\beta }\,, \end{aligned}$$

which satisfies $x_1(s=1)=1$ for any $\beta$ and $k_1$ values. Furthermore, stability analysis of this steady state establishes the following criteria for the existence of an overdamped overshoot response to the step stimulus $s=1$:

6 $$\begin{aligned} \tau /\tau _1 > (1+2\beta )+\sqrt{4\beta (\beta +1)}\,. \end{aligned}$$

Accordingly, the adaptation parameters $\beta \in [0:1]$ and $\tau >1$ control the amplitude and timescale of the overshoot response without changing the steady-state value for $s=1$ (Fig. 2c). More details regarding the relation between the negative-feedback parameters and the adaptation behavior can be found in a previous modeling study^43^.

Before considering a source of variability, we first need to investigate the main effect of transient adaptation dynamics on the fate decision properties. In this case, probabilistic response and fractional killing do not occur, and the system response to a step stimulus is essentially determined by a threshold $s_c$: a stress amplitude *s* greater (resp. lower) than $s_c$ leads to a fate decision toward death (resp., survival). For an adiabatically-slow increase of the stimulus, the system follows the steady-state branch $\vec {x}_{st1}(s)$ of low $x_3$ values before escaping from it for $s>s_{sn}$ through a saddle-node bifurcation. This is not necessarily the case for a step increase of the stimulus, for which transition to death can occur for a stimulus level $s<s_{sn}$ so that $s_{sn}-s_c>0$. The plot of $s_{sn}-s_c$ as function of adaptation parameters in Fig. 2d shows that $s_c=s_{sn}$ for weak enough or fast enough adaptation, while $s_c$ is below $s_{sn}$ for large enough values of both $\beta$ and $\tau$. These two qualitative regimes manifest, in fact, the existence of distinct instability mechanisms (Fig. 2e). For low values of $\beta$ or $\tau$ and thus a small or no overshoot, the threshold property $s=s_c=s_{sn}$ relates with a dynamical trajectory that is destabilized in the vicinity of a saddle-node instability (lower panel of Fig. 2e). In this scenario, $x_3$ species trigger the fate decision depending on the steady-state value of the adaptive species $x_1$, which carries out the role of a bifurcation parameter. In sharp contrast, for high enough value of both $\beta$ and $\tau$ and thus for a significant overshoot response of $x_1$, the threshold property $s=s_c<s_{sn}$ relates with a dynamical trajectory that collides with a saddle instability (upper panel of Fig. 2e).

In summary, while varying $\beta$ and $\tau$ leads to gradual changes of the amplitude and the timescale of the overshoot adaptation profile, we could identify a threshold boundary in the $\{\beta,\tau \}$ space which separates between a saddle-node and a saddle-collision instability scenario. In the saddle-collision scenario, the threshold value $s_c$ becomes very sensitive to the transient characteristics of the overshoot profile, which suggests that fate decision may also become more sensitive to the sources of cell-to-cell variability that impact transient dynamics.

### Adaptation dynamics promotes heterogeneous cell-fate decisions

**Figure 3:**
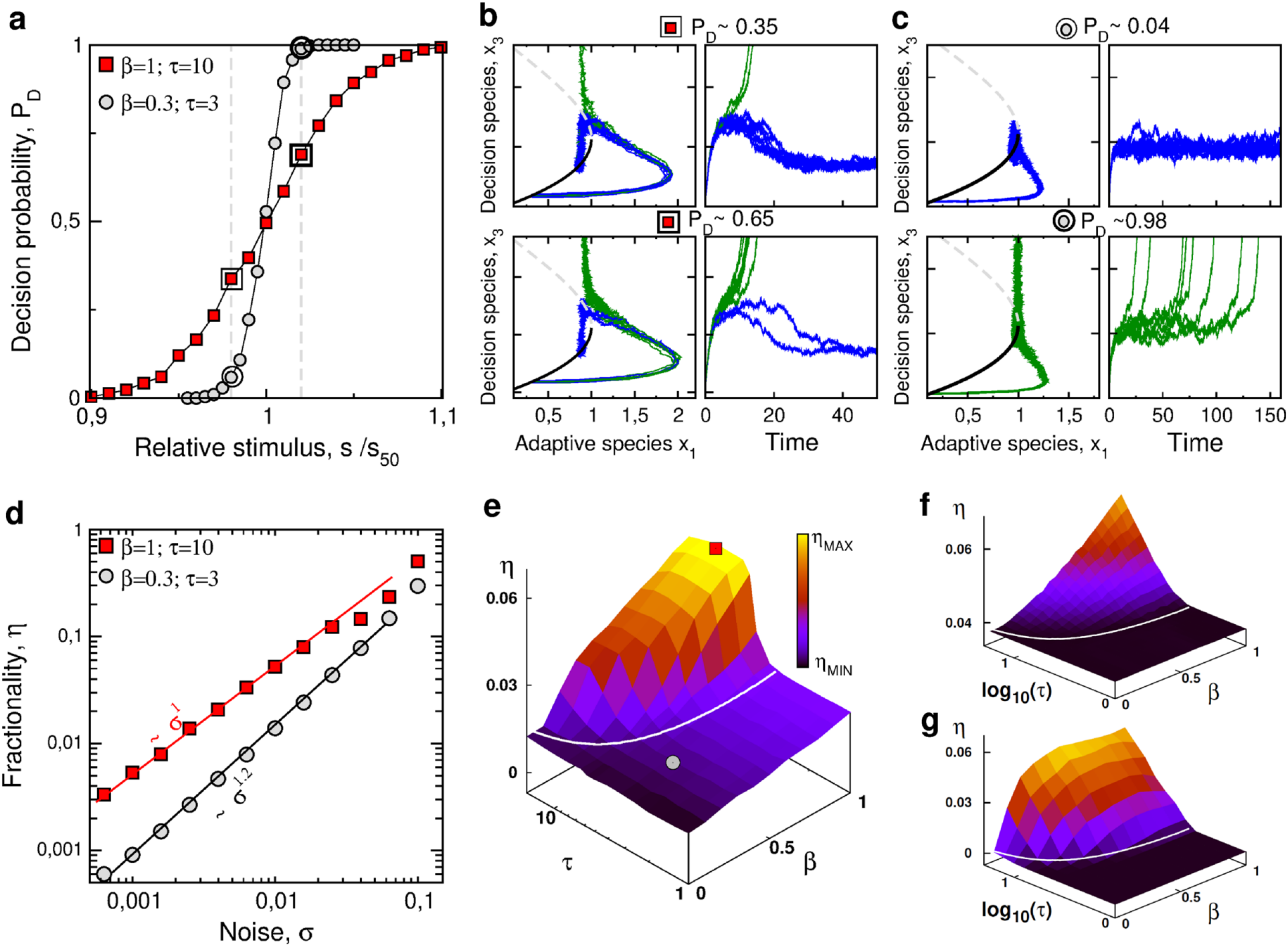
The critical impact of adaptation on cell-fate heterogeneity. Fate decision probability is studied in presence of molecular noise level (**a**–**e**) or other sources of cell-cell variability (**f**–**g**). (**a**) Fate probability curves as function of relative stimulus for the cases of strong/slow adaptation (red squares) and weak/fast adaptation (gray circles). (**b**–**c**) Sample of noisy single-cell trajectories associated with a $\pm 2\%$ change of stimulus level around $s=s_{50}$ (dashed line of panel **a**), which are plotted in the $\{x_1,x_3\}$ state space where steady-state branches $\vec {x}(s)$ are also represented. (**d**) Fractionality index $\eta$ as function of noise with their asymptotic scaling exponents. (**e**) Fractionality index $\eta$ as a function of adaptation parameters $\tau$ and $\beta$ for molecular noise level $\sigma =0.01$. White line delimits the parameter domains of saddle-collision and saddle-node transition scenario (redrawn from Fig. 2c). Red squares ($\beta =1$ and $\tau =10$) and grey circles ($\beta =0.3$ and $\tau =3$) correspond to the two archetypical parameter sets associated to each scenario, which are compared in panels **a**–**d**. (**f**–**g**) Fractionality index $\eta$ as function of $\beta$ and $\tau$ for two sources of cell-cell variability: (**f**) a uniform distribution of stimulus exposure $s^l$ with $\langle \delta s^l \delta s^{l'} \rangle =0.01 \delta _{l,l'}$; (**g**) a uniform distribution of initial conditions $\vec {x}_{st1}(s=0)+ \vec {\delta x}$ with $\langle \delta x_i^l \delta x_j^{l'} \rangle =0.1 \delta _{i,j}\delta _{l,l'}$.

Based on our comprehensive analysis of the deterministic decision dynamics in the coarse-grained model combining adaptation and bistability, we aim to investigate how adaptation influences cell-fate heterogeneity in a population of noisy cells. We therefore apply the general stochastic modeling framework to this biochemical network model (see "Methods" section) and perform a systematic analysis of the probabilistic properties in the $\{\beta,\tau \}$ parameter space and for several noise sources and levels (Fig. 3).

To begin with, we consider the case of cell-to-cell variability arising from molecular noise solely ($s^l=s$ and $\vec {k}^l=\vec {k}$) and focus on the two archetypical parameter sets depicted in Fig. 2 that are associated with weak/fast adaptation ($\beta =0.3$ and $\tau =3$) and strong/slow adaptation ($\beta =1$ and $\tau =10$), respectively. Simulation of Langevin equations for 2000 trials shows that, for the same level of noise, strong/slow adaptation leads to probabilistic response associated with a much larger stimulus range and a much smaller derivative at $P=0.5$ (Fig. 3a). These differences are quantified by the fractionality index $\eta$ that is about four-fold larger for strong adaptation ($\eta \approx 0.05$) as compared to weak adaptation ($\eta \approx 0.012$). To illustrate how adaptation may amplify noise to generate more heterogeneous fate response, we plot the noisy single-cell trajectories in the two scenarios associated with the noise level and the same relative change of stimulus level. When adaptation is strong and slow enough, noisy trajectories remain within some neighborhood of the noise-free trajectory until diverging from it toward different fates when approaching the saddle fixed point, with a slight change of respective fate probability when the stimulus increases (Fig. 3b). This is in sharp contrast with the case of weak (or no) adaptation for which noisy trajectories reach first the neighborhood of a stable fixed point, before eventually escaping over the saddle fixed point toward the other fate when the stimulus slightly increases (Fig. 3c). The qualitative difference between these two stochastic decision scenario is confirmed by the distinct scaling laws $\eta \propto \sigma ^b$, where $b=1$ for strong/slow adaptation and $b \approx 1.2$ for weak/fast adaptation (Fig. 3d). This body of evidences strongly suggest that adaptation dynamics promotes cell-fate heterogeneity, mostly by changing the underlying nonlinear mechanism of decision-making. This is confirmed by the plot $\eta =f(\beta ,\tau )$ (Fig. 3e), which unambiguously shows a qualitative increase of fractionality index $\eta$ specifically in the parameter domain where the saddle-collision scenario occurs (above the white boundary).

Besides molecular noise, other sources of cell-to-cell variability have been tested, such as stimulus exposure or sensitivity $s^l$ (Fig. 3f) or initial conditions $\vec {x}^l(t_0)$ (Fig. 3g). Again, a qualitative increase of $\eta$ is observed in the parameter region associated with a saddle-collision scenario (above the white boundary), though the extent of such increase is much more important for the case of variable initial conditions. This is because variability of initial conditions impacts only transient dynamics, not steady state, while variability of $s^l$ impacts steady-state properties. Adaptation dynamics can promote cell-fate heterogeneity in a qualitative manner, but to varying extent depending on the source of variability and the time profile of the overshoot.

### Adaptation dynamics contributes to fractional killing in an apoptosis model

**Figure 4:**
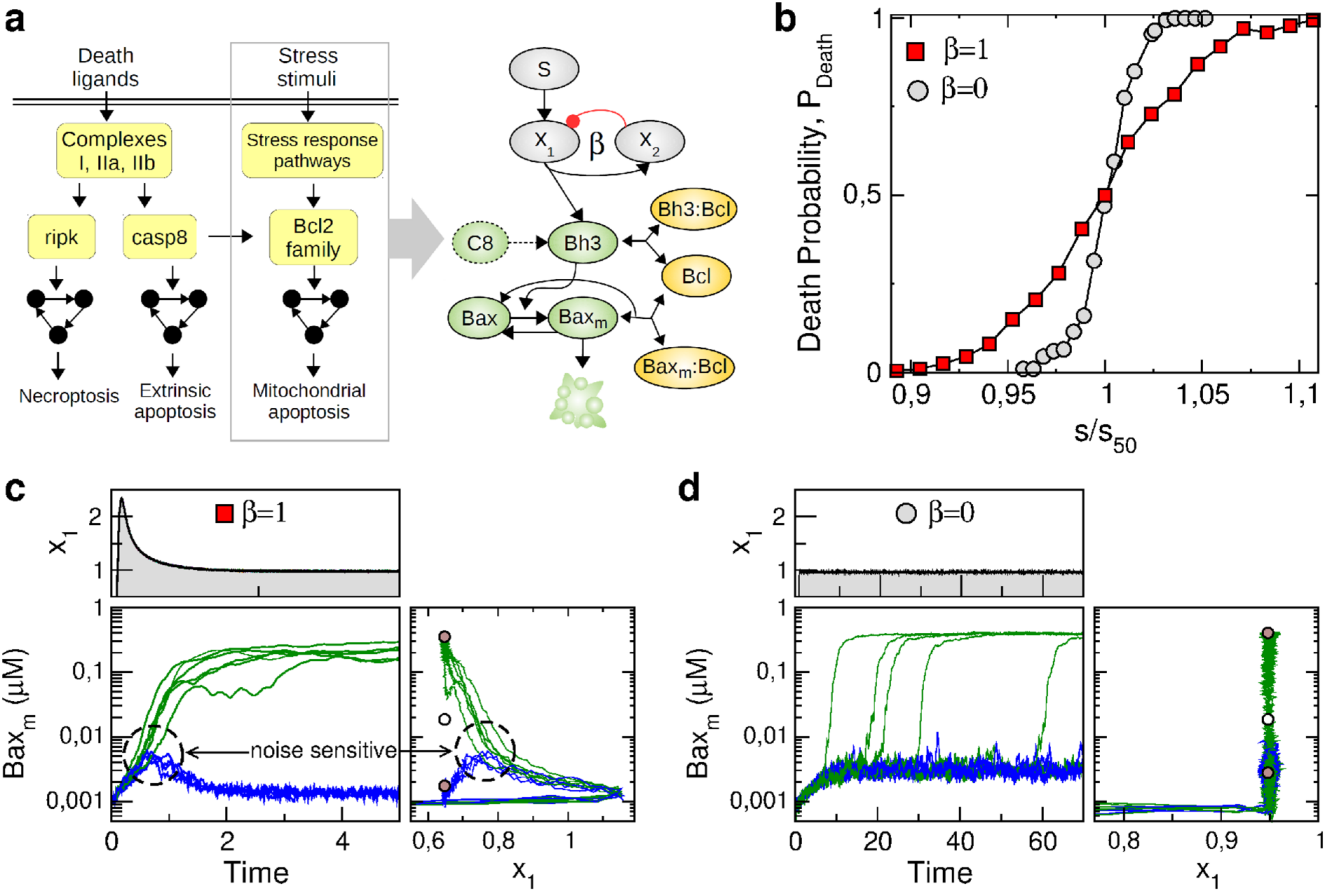
Adaptation-dependent fractional killing in an apoptosis model. (**a**) Some mammalian cell-death pathways associated with fractional killing including the stress-induced mitochondrial pathway of apoptosis (left panel). The detailed model of this study couples the coarse-grained model of stress-induced adaptation module (Eqs. 4a, b) and a published model of the mitochondrial apoptosis initiation module^24^ (right panel). (**b**) Death probability as function of the relative stimulus level $s/s_{50}$ obtained through numerical simulation of Eq. (1) with $\sigma =0.002$, where $\eta$ is about four-fold higher with adaptation ($\beta =1$) compared to without ($\beta =0$). (**c**–**d**) Temporal trajectories of $x_1$ and $\hbox {Bax}_m$ in the presence or the absence of adaptation (**c**: $\beta =1$; **d**: $\beta =0$). Adaptation timescale is set to $\tau =1.25\mathrm {hr}$ to match with the timescale of the apoptotic switch (time unit is hour). Right panels show a 2D state-space projection of the high-dimensional dynamics with respect to the stable and saddle fixed points (brown and white circles) of the deterministic system.

The nonlinear nature of the adaptation-related amplification of noise effect suggests that this mechanism could be effective regardless the complexity of the network model. In other words, we expect to observe a similar noise amplification behavior in more detailed regulatory network model of stress-induced death fate decision as far as the adaptation dynamics leads to a collision to a saddle instability in the state-space of any dimensions. To check this conjecture, we need to replace the effective one-dimensional model of fate decision by a more realistic high-dimensional model of death fate decision. Fractional killing is commonly observed following many types of stress or death ligands, which may trigger death through different pathways^44,45^ depending on the involved multi-protein signaling complexes, transcriptional factors and other signaling and metabolic cues (left of Fig. 4a). Among these many possibilities, we consider the canonical case where the stress signal and damage species mainly impact the intrinsic mitochondrial pathway of apoptosis through the control of the Bh3 member of Bcl-2 family^46^. An alternative possibility could have been to consider the case of TRAIL-induced apoptosis involving caspase 8-dependent activation of both extrinsic and mitochondrial pathways^20^.

The choice of Bh3-dependent mitochondrial apoptosis is motivated by a previous biochemical model of apoptosis initiation^24^, which exhibited several interesting features for our study. First, the model focuses on the initial stage of intrinsic mitochondrial apoptosis, providing a simple picture of the decision-making process by leaving aside the further stages of effector caspase activation and related apoptosis events as well as the complex crosstalk with other programmed death pathways. Second, the topology and parameters of the model are determined in close relation with biological hypothesis and experimental data in a context of chemical stress response. Last, the topological and dynamical properties of the model are featured with a single positive feedback and a bistable behavior which are fully consistent with the minimal set of ingredients that is needed to implement our adaptation-dependent noise-amplification mechanism. In particular, prior knowledge about the bifurcation properties is very helpful to compare $s_c$ and $s_{sn}$ thresholds and make the connection between the low-dimensional and high-dimensional models.

Therefore, the structure of the detailed model merely consists on the coupling between our above adaptation model (Eq. 4a, b) and the published model of mitochondrial apoptosis initiation^24^ (right of Fig. 4a). Specifically, the adaptive species $x_1$ upregulates the synthesis of a pro-apoptotic BH3-only proteins (e.g., Bad, Bim, Bid), keeping in mind that intracellular stress-signaling pathways impacts the mitochondrial apoptosis pathway at various places^45,46^. Regarding the published apoptosis initiation model, the postranslational interactions between the pro-apoptotic Bh3 and Bax proteins and the anti-apoptotic Bcl-2 proteins implement a positive feedback mechanism. Pro-apoptotic signals are prone to increase the level of free Bh3 proteins with respect to the level of Bh3 proteins bound to Bcl-2. Free Bh3 proteins directly interact with inactive cytosolic Bax proteins, thereby inducing conformational change that leads to their activation and mitochondrial translocation. In turn, the activated mitochondria-localized form of Bax can also bind to Bcl-2, resulting in the release of additional free Bh3 proteins from Bh3-Bcl complexes. For a critical synthesis rate of Bh3 proteins, this positive feedback loop produces a bistable switching behavior via a saddle-node bifurcation from low to high levels of free mitochondrial Bax ($\hbox {Bax}_m$)^24^. Then, high enough levels of $\hbox {Bax}_m$ would typically induce the release of cytochrome C and mitochondrial outer membrane permeabilization (MOMP) followed by the formation and activation of the apoptosome and the execution of apoptosis.

The stochastic dynamics of this regulatory network coupling an adaptation module and an apoptosis module is simulated using again the Langevin formalism of Eq. (1) (see "Methods" section) with fixed $\sigma$. Death initiation event is assumed to occur when $\hbox {Bax}_m$ reaches the neighborhood of the high-level steady-state branch. For large number of simulation trials, death probability can be measured as a function of the stress stimulus level, *s*, for distinct adaptation profiles, here with $\beta =0$ or 1 (Fig. 4b). Simulation results reveal that the presence of adaptation leads to a probabilistic response for a broader range of stimulus, which manifests itself by a lower value of the derivative of $P_{death}$ with respect to $s/s_{50}$ associated to a four-fold higher value of $\eta$. Such significant difference in noise-sensitivity $\eta$ correlates to well distinct types of dynamical trajectories associated with survival-death fate decisions (Fig. 4c–d). For $\beta =1$, the overshoot response of $x_1$ species leads to a transient increase of $\hbox {Bax}_m$ during which trajectories finally diverge from each other toward the survival or death attractor (Fig. 4c). This decision is made when approaching the unstable saddle equilibria along its stable manifold (right panel of Fig. 4c). For $\beta =0$, the level of $\hbox {Bax}_m$ reaches and fluctuates around a steady state of low values, before an eventual noise-induced switch toward the death state by escaping over the saddle instability along its unstable manifold (Fig. 4d).

The results obtained with a detailed model of apoptosis are thus consistent with those obtained with the model Eq. (4) involving a minimal decision module (Fig. 4b similar to Figs. 3a and 4c–d similar to Fig. 3b–c). To obtain a similar behavior, it should be noted that (i) adaptation and decision timescales had to be adjusted to each other in the detailed model such that the decrease of $x_1$ during the overshoot profile occurs before $\hbox {Bax}_m$ reaches its upper-branch steady state, and that (ii) the molecular noise impacts more the death species than the adaptive species (compare molecular noise of $Bax_m$ and $x_1$ in Fig. 4c, d). A further step would thus be to check whether these two conditions are fullfiled in the detailed modeling of both the specific signaling pathways producing adaptation at the level of damage-repair pathways^47^, stress-response patwhays^24^ or death-ligand pathways^25,26^, and of the specific death-regulatory pathways that are triggered by these diverse death-inducing stimuli. In these various cases, adaptation and fate decision processes are prone to be implemented by slightly different regulatory network topologies which may modulate the timescale and stochastic characteristics of the dynamical response and influence the extent of the adaptation-dependent fractional killing.

### Theoretical description of stochastic decision properties

**Figure 5:**
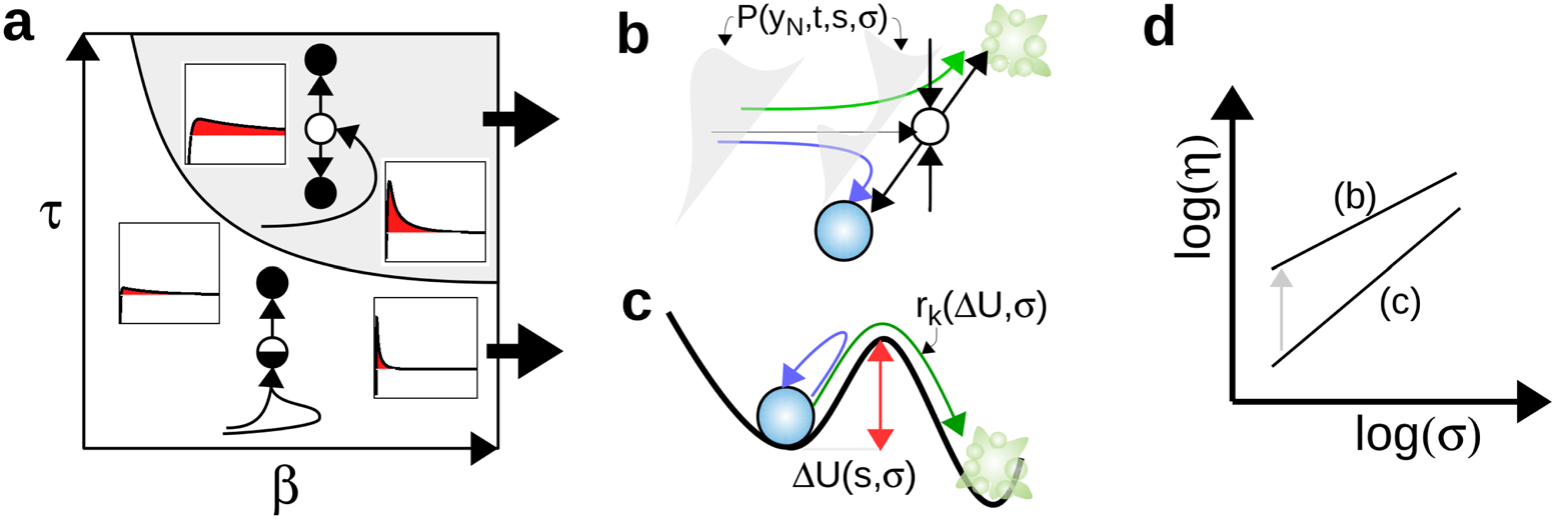
From deterministic to stochastic properties of two distinct cell-fate decision scenarios. (**a**) Deterministic decision mechanims in the space of adaptation parameters. (**b**–**c**) Corresponding stochastic decision mechanisms. (**d**) Qualitative change of fractionality index.

We have shown that the sensitivity of cell-fate decision to molecular noise depends on the state-space paths taken to reach a saddle instability, along, either, its stable manifold or its unstable manifold (Fig. 5a). In order to get further insights into the stochastic nonlinear dynamics involved in this process, we develop a perturbation approach in the limit of small noise and small stimulus changes for which specific scaling laws $\eta =a \sigma ^b$ have been obtained (Fig. 3d). Scaling analysis near instabilities is a common approach to characterize qualitative dynamical behaviors as function of noise, timescales and bifurcation parameters (see textbook^42^ or some case study^48,49^).

In the case of a saddle-collision scenario, perturbed trajectories evolve in the neighborhood of the deterministic trajectory $\vec {x}_c(t,s_c)$ that connects the initial condition $\vec {x}_c(t=0)=\vec {x}_{st1}(s=0)$ and the saddle fixed point $\vec {x}_c(t\rightarrow \infty )=\vec {x}_{sad}(s_{c})$ (Figs. 2d and 3d) and, thus, live on the stable manifold of this saddle $\mathcal{W}^s(\vec {x}_{sad})$ that separates the different fate attractors. Along this singular deterministic trajectory, some local Lyapunov stability exponents (i.e., time-dependent eigenvalues of the Jacobian matrix $J(\vec {x}_c(t))$) become positive such as to amplify transverse perturbations due to molecular noise or heterogeneous initial conditions. Mathematically speaking, linearization of Eq. (1a) about $\vec {x}_c(t)$ defines a class of Langevin equations for the perturbed trajectories $\vec {y}(t)=\vec {x}(t,s_c+\delta s,\sigma )-\vec {x}_c(t)$ whose solution can be decomposed as $\vec {y}(t)=\delta s\,\vec {y}_{\delta s}(t)+\sigma \,\vec {y}_{\sigma }(t)$ where

7 $$\begin{aligned} \vec {y}_{\delta s/\sigma }(t) = \int _0^t\,\Pi (t,t')\,\vec {b}_{\delta s/\sigma }(t')dt'\,. \end{aligned}$$

$\Pi (t,t')$ is the principal fundamental matrix and $\vec {b}_{\delta s/\sigma }$ are the normalized stimuli and noise perturbation vectors given by:

8a $$\begin{aligned} b_{\delta s,i}(t)&= \sum _j \nu _{ji} \frac{\partial a_{j}}{\partial s}(\vec {x}_c(t)) \end{aligned}$$

8b $$\begin{aligned} b_{\sigma ,i}(t)&= \sum _j \nu _{ji}\sqrt{a_{j}(\vec {x}_c(t))} \tilde{\xi }_j(t)\,. \end{aligned}$$

To compute the fractionality index $\eta$, the key statement is that fate decisions (resp., probability) are determined by the deviation (resp., distribution) of trajectories onto the normal direction $y_N$ of the $(N-1)$-dimensional stable manifold of the saddle. The mean and variance of the normal distribution $P(y_N,t)$ evolves in time until the decision time $t^*$ at which the distribution splits and many trajectories leave the neighborhood of $\vec {x}_c(t)$ (Fig. 5b). Rewriting Eq. (2) as $P_D=\int _{\mathbb {R}^+} P(y_N,t^*)dy_N$ and decomposing $\frac{dP_D}{ds}=(\frac{dP_D}{d \langle y_N\rangle})(\frac{d\langle y_N\rangle}{ds})=(2\pi \sigma ^2\langle y_{N,\sigma }^2\rangle)^{-1/2}(\langle y_{N,\delta s}\rangle)$, we can derive the following expression for $\eta$:

9 $$\begin{aligned} \eta =\sqrt{\frac{\pi }{2}}\frac{\sqrt{\langle y_{N,\sigma }(t^*)^2\rangle }}{s_{50}\langle y_{N,\delta s}(t^*)\rangle} \sigma \,. \end{aligned}$$

This formula shows an asymptotic scaling law $\eta \propto \sigma$ (Figs. 3d and 5d) while the prefactor depends in a sophisticated manner on the local stability properties (via $\Pi$) and sensitivity properties (via $\vec {b}$) of the transient trajectory. A similar derivation in the 1D case has been previously performed to show that this scaling law also depends on the speed of the trajectory toward the saddle instability^48^.

The sensitivity to noise is very different in the other scenario of noise-induced escape from a stable state ($\vec {x}_{st1}$) over a saddle barrier ($\vec {x}_{sad}$), which is a very common behavior associated with the escape from metastability^49,50^. For this decision-making regime, an effective potential *U*, a potential barrier $\Delta (s)=U(\vec {x}_{sad}(s))-U(\vec {x}_{st1}(s))$ and a Kramers escape rate $r_K(s) \propto \exp {(2\Delta (s)/\sigma ^2)}$ can be usually defined, even for multi-dimensional systems and multiplicative noise^51^ (Fig. 5c). Given a fate-decision probability $P_D(t) \approx 1-\exp {(-r_Kt)}$, the fractionality index can be derived and approximated as :

10 $$\begin{aligned} \eta = \left( \frac{s_{50}\ln {2}}{r_K} \frac{dr_K}{ds}\right) ^{-1} \approx \frac{\sigma ^2}{(2\ln {2})\,s_{50}\,\partial _s\Delta }\,. \end{aligned}$$

For the one-dimensional model of bistability used in Eq. (4c), the particular scaling relation $s_{50}\,\partial _s \Delta \sim \sigma ^{0.8}$ (as the threshold $s_{50}$ depends on $\sigma$) leads to the scaling law $\eta \propto \sigma ^{1.2}$ obtained in Fig. 3d.

To conclude, these very distinct formulas for $\eta$ highlight that the conversion of intracellular fluctuations into heterogeneous cellular fate response sharply differ depending on the transition scenario. The saddle-collision scenario is characterized with the amplification of small perturbations due to the local instability of trajectories when approaching the saddle state during the overshoot of decision variables (e.g., $x_3$ or $\hbox {Bax}_m$). In contrast, the more common scenario of a noise-induced escape from a metastable state does not display this amplification mechanism, while the transition rate $r_K$ is very sensitive to stimulus level due to the exponential-like dependency on the saddle barrier height.

## Discussion

The present modeling study deciphers the role of adaptation dynamics in promoting cell-fate heterogeneity associated for instance with the fractional killing behavior. A common property of adaptation is the transient overshoot of some cellular variables above its steady state value, which can be implemented by diverse circuit topologies^32^ and which is subjected to tradeoffs associated with homeostatic or sensory process^33,34,52,53^. In addition, we propose that this transient overshoot dynamics can also significantly impact fate-switching behaviors, so as to extend the stimulus range of fate heterogeneity and to allow for tunable fate probability. This adaptation-dependent fate stochasticity relies on the manner how the overshoot of some intracellular species drive cell state in the neighborhoohd of a saddle instability, rather than a saddle-node instability, along a path where molecular noise are more prone to promote divergent decisions. This noise-amplification behavior illustrates how molecular noise and instability mechanisms can cooperate to shape cellular dynamics, like genetic timers^54^, boundary formation^55^ or versatile sensory processing^56^.

The biological relevance of the proposed mechanism is most likely in a context of fractional killing for which the choice between life and death depends on adaptation processes. The timescales of those adaptation responses range from half an hour to few hours depending on stress type and regulatory mechanisms^47,57,58^ which is of the range of magnitude of the initiator caspase rise time and death onset timing. Moreover, noise-induced fate heterogeneity is the most effective when fluctuating variables are those involved in the positive feedback that triggers death initiation. This requirement is consistent with modeling evidences that variability in diverse regulatory molecules can contribute in very different ways to variability in cell death outcomes^20^ and that the main contributions seem to occur in the initial decision commitement phase, whether it is at the level of the fluctuations of short-lived antiapoptotic proteins^22^ or the stochastic assembly of DISC/RIPoptosome platform^23^. The manner how cell fate is determined by the impact of these fluctuations at the level of concentration trajectories has been also investigated^24,25,27^. In relation to these studies, our study presents a broad and comprehensive view of this cooperative process and, thus, provides strategies, by monitoring transient characteristics, to either increase or reduce fractional killing.

The profile characteristics of adaptation dynamics, such as the ratio between its maximal and steady-state values, are highly sensitive to the temporal profile of the stimulus. Ramp increase of a stimulus or a preconditioning stimuli are known to reduce the transient overshoot behavior. This feature has been exploited to test the role of adaptation in oxidative stress response of yeast^59^, the osmotic stress response of yeast^60^ or ethanol stress in Bacillus^61^. In case of stress-induced fate response, monitoring the stress stimulus profile would therefore be expected to modulate not only threshold stimulus level ($s_{50}$), but also the degree of heterogeneity of the response ($\eta$). This provides a practical mean to test the role of adaptation for cell-fate heterogeneity, and to design dose delivery protocols of treatment to cope with fractional killing of cancer cells or microbial organisms.

It is tempting to extrapolate the biological relevance of such adaptation-dependent mechanism beyond the scope of fractional killing and transient adaptation dynamics. The mechanism itself only requires a regulatory network featured with an upsteam overshoot response and a downstream switching response, which could be implemented by diverse network topologies and in diverse cell-fate contexts. For instance, overshoot dynamics can also occur in regulatory systems comprising incoherent feedforward loops^32^, but also in excitable or pulsatile systems combining negative and positive feedback loops. For the latter case, numerous signaling pathways, such as P53, Erk or NF-$\kappa$B, display a versatile pulsatile dynamics, which has been proposed to expand signal-processing capabilities and determine cell fate accordingly^14,15^. In relation to our result, the transient and stochastic characteristics of these signaling dynamics may also suggest a role for promoting cell-fate heterogeneity. This is supported by some experimental evidences that have mapped the cell-cell variability of the pulsing dynamics of Erk^10,11^, p53^62,63^, $\beta$-catenin^13^ and NF-$\kappa$B^12^ with the heterogeneity of cell-fate outcomes. Data-driven and fine-grained modeling of specific dynamic signaling and fate-regulatory pathways^11,20,62,64^ are definitively the step further to evaluate on a case-by-case basis to which extent transient adaptation or pulsing dynamics may contribute, fortuitously or functionally, to cell-fate heterogeneity.

## Methods

### Coarse-grained model

The set of regulatory reactions depicted in Fig. 3a consists in the following basal/regulated synthesis terms and basal/regulated degradation terms:

11a $$\begin{aligned}&\overset{b_1}{\rightarrow } X_1 \,\,;\,\, \overset{b_{S1}S}{\rightarrow } X_1 \,\,;\,\, \overset{b_2}{\rightarrow } X_2 \,\,;\,\, X_1 \overset{b_{12}}{\rightarrow } X_2+ X_1 \,\,;\,\, X_1 \overset{b_{13}}{\rightarrow } X_3+X_1 \,\,;\,\, \xrightarrow {b_{33}f_h(X_3,K_3)} X_3 \end{aligned}$$

11b $$\begin{aligned}&X_1+ X_2 \overset{d_1}{\rightarrow } X_2 \,\,;\,\, X_2 \overset{d_2}{\rightarrow } \oslash \,\,;\,\, X_3 \overset{d_3}{\rightarrow } \oslash \,, \end{aligned}$$

which can be translated into a system of differential equations using the law of mass action :

12a $$\begin{aligned} \dot{X_1}&= b_{1} + b_{S1}S - d_1 X_2 X_1 \,, \end{aligned}$$

12b $$\begin{aligned} \dot{X_2}&= b_2 + b_{12} X_1 - d_2 R \,, \end{aligned}$$

12c $$\begin{aligned} \dot{X_3}&= b_{13} X_1 + b_{33}\frac{X_3^2}{K_3 + X_3^2} - d_3 X_3\,. \end{aligned}$$

To obtain the set of Eq. (4), we perform a nondimenzionalization procedure to reduce the number of parameters and to define effective parameters that control separately different features of the dynamics such as response timescales, transient nonlinear response and steady states. Accordingly, we have introduced dimensionless time $\tilde{t}$, concentration $x_i$ and stimulus *s* and defined rescaling variables ($X_{i,0}$, $S_0$) and aggregate parameters ($\tau _i$, $\beta$ and $k_i$), as the following:

13a $$\begin{aligned}&\tilde{t}=t\,d_3 \,\,; \,\, x_i=X_i/X_{i,0} \,\,; \,\, s=S/S_0 \end{aligned}$$

13b $$\begin{aligned}&X_{1,0}=\frac{k_2 d_3 X_{3,0}}{b_{13}} \,\,; \,\, X_{2,0}=\frac{b_{12}X_{1,0}+b_2}{d_2} \,\,; \,\, X_{3,0}=\frac{b_{33}}{d_3} \,\,; \,\, S_{0} = \frac{d_1X_{1,0}X_{2,0}-b_1}{b_{S1}} \end{aligned}$$

13c $$\begin{aligned}&\tau =\frac{d_3}{d_2} \,\,; \,\, \tau _1=\frac{d_3}{d_1X_{2,0}} \,\, ; \,\, k_1 = 1-\frac{b_1}{d_1X_{1,0}X_{2,0}} \,\, ;\,\, \beta =\frac{b_{12}X_{1,0}}{d_2 X_{2,0}} \,\, ;\,\, k_3=\frac{K_3}{X_{3,0}^2} \,. \end{aligned}$$

These changes of variables and parameters simplify Eq. (12) into Eq. (4) where dimensionless time $\tilde{t}$ is noted *t* again for simplicity. The chemical Langevin equation associated to Eq. (4) is also characterised with a rescaled noise $\sigma =(\Omega d_3 X_0)^{-1/2}$ where $\Omega$ is the system size and $X_{i,0} \equiv X_0 \, \forall i$. Finally, reaction rates and stoichiometry matrices are given by:

14 $$\begin{aligned} \vec {a}=\left[ \frac{1-k_1}{\tau _1} ,\, \frac{k_1 s}{\tau _1} ,\, \frac{x_1 x_2 }{\tau _1} ,\, \frac{1-\beta }{\tau } ,\, \frac{\beta x_1}{\tau } ,\, \frac{x_2}{\tau } ,\, k_2 x_1 ,\, \frac{k_3 x_3^2}{k_4+x_3^2} ,\, x_3 \right] ^T; \quad \nu = \left[ \begin{array}{ccccccccc} 1 &{} 1 &{} -1 &{} 0 &{} 0 &{} 0 &{} 0 &{} 0 &{} 0 \\ 0 &{} 0 &{} 0 &{} 1 &{} 1 &{} -1 &{} 0 &{} 0 &{} 0 \\ 0 &{} 0 &{} 0 &{} 0 &{} 0 &{} 0 &{} 1 &{} 1 &{} -1 \end{array}\right] \end{aligned}$$

where $k_1=0.9$, $k_2=0.056$, $k_3=1$, $k_4=0.2$, $\tau _1=0.1$, while $\beta$ and $\tau$ are varied.

### Detailed model

Equations and parameters of the biochemical reaction model of apoptosis initiation have been taken from^24^. From the original model, the equations for CIAP, p53 and Mdm2 have been removed and the equation for Bh3T has been changed to incorporate activation by $x_1$ and to display a slower response:

15 $$\begin{aligned} \frac{d[Bh3T]}{dt}=k_{sBH3}+k_{s2}\,x_1+k_{s3} [C8] - k_{dBH3} [Bh3T] \end{aligned}$$

The corresponding Langevin equation (Eq. 1) considers the following state vectors, reaction rate vectors and stoichiometry matrix:

16 $$\begin{aligned} \vec {x}=\left[ x_1 ,\, x_2 ,\, [Bh3T] ,\, [C8] ,\, [BaxmT] ,\, [Baxm:Bcl] ,\, [Bh3:Bcl] \right) ^T \,; \vec {y}=\left[ [BaxT]-x_5 ,\, x_5-x_6 ,\, x_3 - x_7, [BclT]-x_5-x_6 \right] ^T \end{aligned}$$

17a $$\begin{aligned}&a_{1,.,12}= \left[ \frac{1-k_1}{\tau _1} ,\, \frac{k_1 s}{\tau _1} ,\, \frac{x_1 x_2 }{\tau _1} ,\, \frac{1-\beta }{\tau } ,\, \frac{\beta \,x_1}{\tau } ,\, \frac{x_2}{\tau } ,\, k_{sBh3} ,\, k_{s2}\, x_1 ,\, k_{s3}\, x_4 ,\, k_{dBh3}\, x_3 ,\, k_{aC8} ,\, k_{iC8}\, x_4 \right] ^T \end{aligned}$$

17b $$\begin{aligned}&a_{13,.,20}=\left[ k_{f1}\,y_1 ,\, k_{f2}\,y_3\,y_1 ,\, k_b\, x_5 ,\, k_{asXC}\,y_4\,y_2 ,\, k_{dsXC}\,x_6 ,\, k_{b}\,x_6 ,\, k_{asHC}\,y_4\,y_3 ,\, k_{dsHC}\,x_7\right] ^T \end{aligned}$$

where $$k_{sBH3}=0.025$$, $$k_{s2}=0.02$$ $$k_{s3}=k_{dBH3}=0.25$$, $$k_{aC8}=0.03$$, $$k_{iC8}=0.1$$, $$k_{f1}=1$$, $$k_{f2}=300$$, $$k_{b}=2$$, $$k_{asXC}=9000$$, $$k_{dsXC }=0.05$$, $$k_{asHC}=1000$$, $$k_{dsXC}=0.01$$, $$[BaxT]=1$$ and $$[BclT]=0.85$$.

18 $$\begin{aligned} \nu = \left[ \begin{array}{cccccccccccccccccccc} 1 &\quad{} 1 &\quad{} -1 &\quad{} 0 &\quad{} 0 &\quad{} 0 &\quad{} 0 &\quad{} 0 &\quad{} 0 &\quad{} 0 &\quad{} 0 &\quad{} 0 &\quad{} 0 &\quad{} 0 &\quad{} 0 &\quad{} 0 &\quad{} 0 &\quad{} 0 &\quad{} 0 &\quad{} 0 \\ 0 &\quad{} 0 &\quad{} 0 &\quad{} 1 &\quad{} 1 &\quad{} -1 &\quad{} 0 &\quad{} 0 &\quad{} 0 &\quad{} 0 &\quad{} 0 &\quad{} 0 &\quad{} 0 &\quad{} 0 &\quad{} 0 &\quad{} 0 &\quad{} 0 &\quad{} 0 &\quad{} 0 &\quad{} 0 \\ 0 &\quad{} 0 &\quad{} 0 &\quad{} 0 &\quad{} 0 &\quad{} 0 &\quad{} 1 &\quad{} 1 &\quad{} 1 &\quad{} -1 &\quad{} 0 &\quad{} 0 &\quad{} 0 &\quad{} 0 &\quad{} 0 &\quad{} 0 &\quad{} 0 &\quad{} 0 &\quad{} 0 &\quad{} 0 \\ 0 &\quad{} 0 &\quad{} 0 &\quad{} 0 &\quad{} 0 &\quad{} 0 &\quad{} 0 &\quad{} 0 &\quad{} 0 &\quad{} 0 &\quad{} 1 &\quad{} -1 &\quad{} 0 &\quad{} 0 &\quad{} 0 &\quad{} 0 &\quad{} 0 &\quad{} 0 &\quad{} 0 &\quad{} 0 \\ 0 &\quad{} 0 &\quad{} 0 &\quad{} 0 &\quad{} 0 &\quad{} 0 &\quad{} 0 &\quad{} 0 &\quad{} 0 &\quad{} 0 &\quad{} 0 &\quad{} 0 &\quad{} 1 &\quad{} 1 &\quad{}-1 &\quad{} 0 &\quad{} 0 &\quad{} 0 &\quad{} 0 &\quad{} 0 \\ 0 &\quad{} 0 &\quad{} 0 &\quad{} 0 &\quad{} 0 &\quad{} 0 &\quad{} 0 &\quad{} 0 &\quad{} 0 &\quad{} 0 &\quad{} 0 &\quad{} 0 &\quad{} 0 &\quad{} 0 &\quad{} 0 &\quad{} 1 &\quad{}-1 &\quad{}-1 &\quad{} 0 &\quad{} 0 \\ 0 &\quad{} 0 &\quad{} 0 &\quad{} 0 &\quad{} 0 &\quad{} 0 &\quad{} 0 &\quad{} 0 &\quad{} 0 &\quad{} 0 &\quad{} 0 &\quad{} 0 &\quad{} 0 &\quad{} 0 &\quad{} 0 &\quad{} 0 &\quad{} 0 &\quad{} 0 &\quad{} 1 &\quad{}-1 \end{array}\right] \end{aligned}$$

### Numerical simulation and dynamical analysis

For both models, numerical integation of Langevin equations are performed with 4th-order Runge-Kutta method and probability distribution $P_D(s)$ are plotted with a statistics of 2000 trials with a measurement time of $t^*=500$. $\eta$ is computed by interpolating $P_D(s)$ and approximating $\partial _s P_D(s_{50}) \approx \frac{0.4}{s_{70}-s_{30}}$ where $P_D(s_x)=x/100$. State-space trajectories are represented in some relevant subspace of the state space where the steady states $\vec {x}_{st/sad/sn}$ satisfying $f(\vec {x})=0$ are represented by the conventional filled/empty/half-empty circles. The steady-state branches $\vec {x}_{st/sad}(s)$ are also represented for the sake of comparison for different parameter values. The set of mathematical notations used are given in the Table 1.
